# Trauma Research Funding: An Emergency in Emergency Care

**DOI:** 10.7759/cureus.67748

**Published:** 2024-08-25

**Authors:** Robert Goodwin, Sarthak Parikh, Christopher Hendrix, Brent Norris, Mani Cheruvu

**Affiliations:** 1 Trauma Institute, Saint Francis Health System, Tulsa, USA; 2 Graduate Medical Education and Center for Clinical Research and Sponsored Programs, Saint Francis Health System, Tulsa, USA; 3 Department of Orthopaedic Surgery, Oklahoma State University, Tulsa, USA; 4 Orthopaedics and Trauma, Orthopaedic and Trauma Service of Oklahoma, Tulsa, USA

**Keywords:** years of potential life lost (ypll), national institutes of health (nih), centers for disease control and prevention (cdc), healthcare disparities, research funding, trauma

## Abstract

Introduction: Trauma remains a significantly underfunded area of medical research despite its status as a leading cause of death and substantial economic burden in the United States. This study explores the disparity in trauma research funding, focusing on Oklahoma compared to neighboring and populous states.

Methods: Using data from the Centers for Disease Control and Prevention (CDC)'s Web-based Injury Statistics Query and Reporting System (WISQARS™) and the National Institutes of Health (NIH)'s RePORT databases, we analyzed age-adjusted years of potential life lost (YPLL) due to trauma and corresponding NIH funding across different states and nationally. Statistical analyses included bivariate comparisons and standardization of funding data per population and YPLL.

Results: From 2010 to 2020, NIH allocated approximately $124 billion nationally, with only 5% dedicated to trauma, amounting to $1,772.32 per age-adjusted YPLL. Oklahoma and Kansas exhibited the lowest NIH funding per YPLL compared to New York and California. Funding for the National Injury Prevention and Control, a subdivision of the CDC, has shown significant increases, ranking third in 2022.

Conclusion: This study highlights the urgent need for enhanced trauma research funding to address its disproportionate impact on mortality and healthcare costs. Strategic allocation of resources is essential to advance trauma care and align research priorities with public health needs.

## Introduction

Despite years of advocacy for increased funding, trauma research remains a critically underfunded area of medicine compared to other research fields [1-5]. It is the leading cause of death for people aged 1-44 years across the United States and costs the nation about $671 billion per year. Trauma-related deaths account for about 214,000 patients each year and have continued to increase since 2000, accounting for more deaths than communicable diseases and infectious diseases combined [6].

The sequela following traumatic events not only compromises a patient’s quality of life but also increases the burden on healthcare in the United States [7]. For instance, it has been shown that patients suffering from gunshot wounds have lasting physical, mental, emotional, and social effects beyond the economic and mortality burden [8]. Trauma survivors, like those suffering from gunshot wounds, require extensive ongoing care including rehabilitation services and regular follow-ups with providers. These patients are also subject to the mental, physical, and financial problems associated with their incident which can drain resources from other areas of healthcare.

As rates of trauma continue to increase, so does the need for research. According to the National Institutes of Health (NIH), trauma research ranks last among 27 disease categories in terms of funding [9]. Although this may stem from a variety of factors, limiting trauma research restricts opportunities to improve healthcare. Moreover, as the number of trauma centers and patient volumes increases, trauma funding has not [10]. Therefore, continuous investment in trauma research is imperative to reduce fatalities and improve the overall quality of healthcare in the United States.

The purpose of this study is to analyze and present publicly available data on trauma research funding in Oklahoma, comparing it with neighboring states and with the most populous states in the United States. It is hypothesized that trauma research funding, when analyzed by age-adjusted for years of potential life lost (YPLL), is lower in Oklahoma compared to other nationally funded areas of healthcare. Please note that "funding", in this article, means research funding.

## Materials and methods

NIH funding per YPLL

To analyze the relationship between NIH funding and YPLL for major causes of death, we utilized data from two key sources. First, the Centers for Disease Control and Prevention (CDC)'s Web-based Injury Statistics Query and Reporting System (WISQARS™) provided data on the top 10 causes of death nationally and in Oklahoma from 2010 to 2020 [11]. We combined data on suicide, homicide, and unintentional injury (excluding overdose) into a single category termed "Trauma" and recorded the age-adjusted YPLL for each cause of death for both the United States and Oklahoma.

Next, we accessed the Research Portfolio Online Reporting Tools (RePORT) database to obtain NIH funding data [12]. We identified funded research projects corresponding to the top 10 causes of death listed in the WISQARS data. To capture funding information, we used predefined search criteria and keywords related to each cause of death. We then extracted and summed the total funding amounts, including both awards and sub-awards (e.g., R01 grants and fellowships) for each category.

Funding data was categorized into national totals and state-specific totals, with a particular focus on the two most populous states (New York and California) and neighboring states to Oklahoma (Arkansas, Missouri, and Kansas). To make funding comparisons more meaningful, we standardized the total funding amounts by dividing them by the population figures from the 2020 Census, and then multiplying by 100,000. To further standardize the data, we divided the standardized funding amount by the age-adjusted YPLL, allowing for comparison across the United States, Oklahoma, and the selected states.

CDC funding over time

We reviewed the CDC Congressional Justifications for the years 2010 through 2022, accessible via the CDC Budget webpage [13], to gather data on funding allocations across six major categories each year. To ensure comparability over time, we adjusted the funding amounts for inflation using the annual Consumer Price Index (CPI) data from the United States Bureau of Labor Statistics.

Given the straightforward nature of the calculations involved, we relied on simple arithmetic methods to analyze the data, with no advanced statistical tests employed.

All data used in this study are publicly available, so no specific permissions were required for access. According to CDC and NIH definitions, "trauma" encompasses severe injuries resulting from accidents, falls, violence, and other external forces, including motor vehicle crashes, falls, and assaults.

## Results

NIH funding per YPLL

From 2010 to 2020, total NIH funding for the top 10 causes of death in the United States amounted to approximately $124 billion, which translates to about $37 million per 100,000 people (Table 1). Of this total, approximately 5% was allocated to trauma research. The age-adjusted YPLL for trauma research nationwide was 1,623 per 100,000 people, making it the highest among all causes of death.

**Table 1:** Funding and age-adjusted years of potential life lost by cause of death nationally and in Oklahoma, 2010-2020 *Encompasses trauma, excluding overdose AA YPLL: age-adjusted years of potential life lost

Cause	Total NIH funding including sub-awards	Adjusted funding per 100,000	AA YPLL per 100,000	Funding per AA YPLL	Percentage of funding per YPLL
United States					
Suicide	$1,846,539,84	$554.66	344.9	$1.61	0%
Homicide	$16,738,556.00	$5,027.88	229.2	$21.94	0%
Unintentional Injury*	$6,106,620,690.00	$1,834,287.84	1048.9	$1,748.77	1%
Congenital Anomalies	$2,968,708,885.00	$891,731.59	158.5	$5,626.07	4%
Perinatal Period	$6,704,731,680.00	$2,013,946.57	282.1	$7,139.12	6%
Diabetes Mellitus	$2,968,708,885.00	$891,731.59	94.5	$9,436.31	7%
Heart Disease	$21,748,030,895.00	$6,532,606.26	468.5	$713,943.66	11%
Liver Disease	$7,503,874,864.00	$2,253,990.72	116.9	$19,281.36	15%
Malignant Neoplasms	$66,275,725,025.00	$19,907,697.31	555.3	$35,850.35	28%
Cerebrovascular	$9,692,287,318.00	$2,911,339.29	79.8	$36,482.95	28%
Total	$123,987,273,337.84	$37,242,913.70	3378.6	$129,532.14	100%
Oklahoma					
Suicide	$0.00	$0.00	504	$0.00	0%
Homicide	$0.00	$0.00	266	$0.00	0%
Heart Disease	$91,957,014.00	$2,322,146.82	767	$3,027.57	10%
Unintentional Injury*	$17,366,531.00	$438,538.76	1221.5	$359.02	1%
Congenital Anomalies	$4,441,303.00	$112,154.12	219	$512.12	2%
Liver Disease	$6,769,353.00	$170,943.26	166	$1,029.78	3%
Perinatal Period	$12,381,043.00	$312,652.60	317	$986.29	3%
Heart Disease	$91,957,014.00	$2,322,146.82	767	$3,027.57	10%
Malignant Neoplasms	$155,703,265.00	$3,931,900.63	715	$5,499.16	17%
Diabetes Mellitus	$85,750,802.00	$2,165,424.29	140	$15,467.32	49%
Total	$393,992,038.00	$9,949,293.89	4422.5	$31,512.32	100%

Nationally, NIH funding for trauma was $1,772.32 per age-adjusted YPLL, representing only 1% of the total funding. This was the lowest funding rate per age-adjusted YPLL among the categories studied. Funding levels in Oklahoma were similar to the national average. However, when compared with funding in New York, California, Arkansas, Missouri, and Kansas, Kansas and Oklahoma had the lowest trauma funding per YPLL, with amounts of $23.40 and $24.68, respectively (Table 2).

**Table 2:** Trauma funding and age-adjusted years of potential life lost across states, 2010-2020 AA YPLL: age-adjusted years of potential life lost

State	Funding total	Population	Funding per 100,000	AA YPLL	AA YPLL per 100,000	Funding per AA YPLL
Kansas	$9,405,971.00	2,937,847	$320,165.45	402,040	13,685	$23.40
Oklahoma	$17,366,531.00	3,959,353	$438,548.76	703,477	17,767	$24.68
Arkansas	$21,907,173.00	3,011,524	$727,444.74	505,744	16,794	$43.32
Missouri	$116,702,548.00	6,154,913	$1,896,087.69	1,102,056	17,905	$105.90
California	$834,361,870.00	39,538,223	$2,110,266.49	3,933,202	9,948	$212.13
New York	$567,660,373.00	20,201,249	$2,810,026.12	1,774,784	8,786	$319.85

CDC funding over time

**Figure 1:**
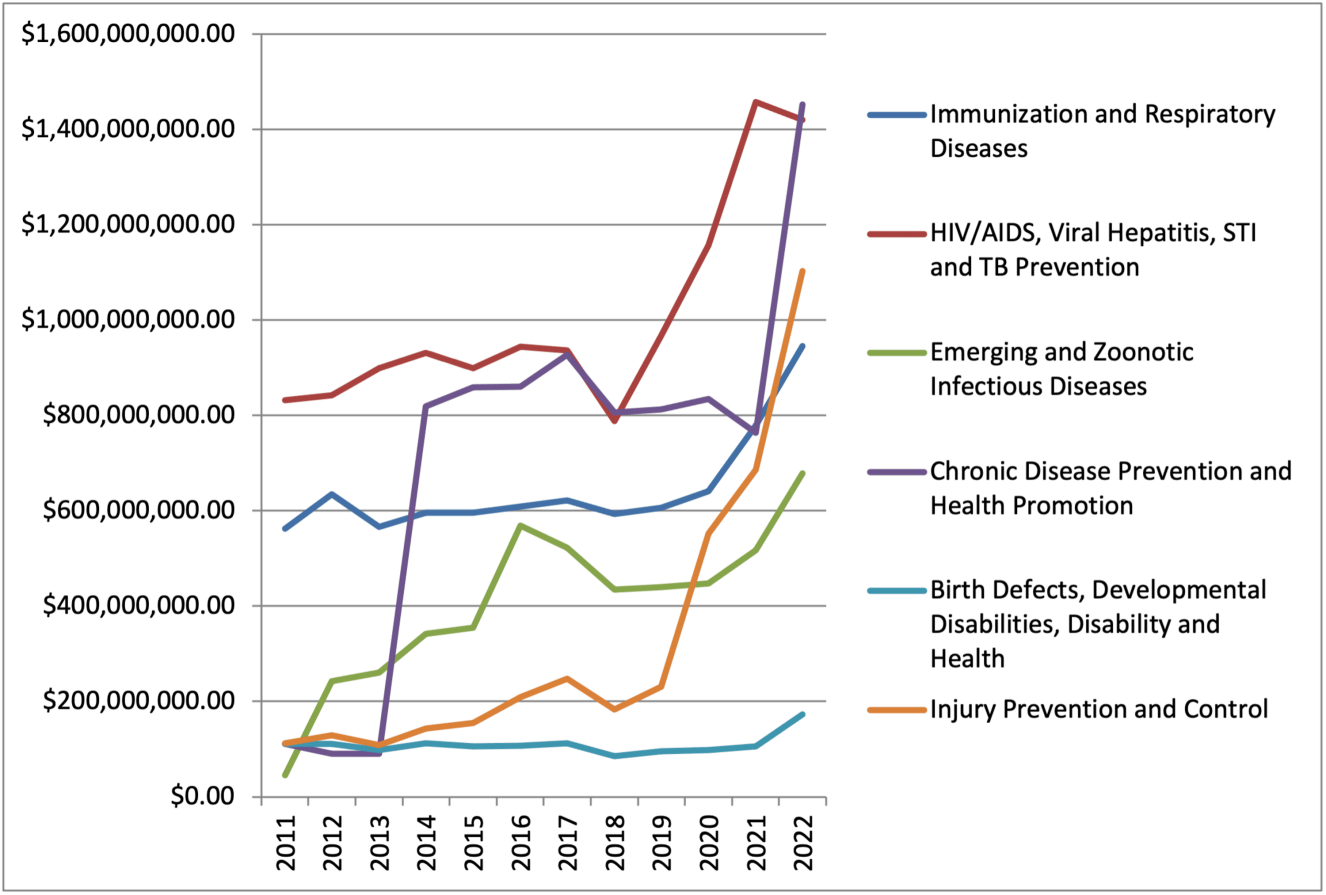
CDC funding by category, 2011-2022 STI: sexually transmitted infections; TB: tuberculosis; CDC: Centers for Disease Control and Prevention

The CDC’s "Injury Prevention and Control" category, which encompasses trauma care, prevention, and research, has experienced significant growth in funding from 2019 to 2022. By 2022, this category was ranked third in funding priorities, following HIV/AIDS, viral hepatitis, sexually transmitted infections (STIs) and tuberculosis (TB) prevention, and chronic disease prevention and health promotion (Figure 1).

It is important to note that NIH funding for suicide and homicide, two key components of the Trauma category, was absent in Oklahoma. Moreover, both the standardized funding amount and the age-adjusted funding amount have increased significantly since 2015, contributing to improvements in life expectancy in the United States.

## Discussion

The aim of this study was to compare trauma funding data in Oklahoma with national data and states having both similar and contrasting demographics. It was hypothesized that age-adjusted trauma research funding is lower in Oklahoma compared to other nationally funded areas of healthcare. The age-adjusted YPLL serves as an appropriate metric for comparison between states because it neutralizes population variations among different states, allowing for national comparisons.

According to the results, trauma is the least funded area (when accounting for YPLL) by the NIH both at the national and state levels. The CDC has recognized this lack of funding and has increased its contributions in 2019 to make it the third most funded category. When comparing trauma funding in Oklahoma to nearby states and the most populous states, Oklahoma received the second lowest trauma funding per YPLL.

While trauma research may face funding challenges, it is important to contextualize these results by comparing them to the leading causes of death and assessing their value relative to other nationally funded sectors. According to the CDC, accidents (unintentional injury, a subcategory of trauma) were the fourth leading causes of death in 2019 behind heart disease, cancer, and COVID-19 [14]. Provisional data from 2023 ranks it fifth, behind heart disease, malignant neoplasm, cerebrovascular disease, and chronic lower respiratory disease [15]. In 2022 it was ranked third, behind heart disease and malignant neoplasms [16]. Assuming that research funding priority should correlate with the leading causes of death, trauma funding should at the least rank below heart disease, malignant neoplasms, and cerebrovascular accidents in the NIH-funded categories. The data presented in this study illustrates that trauma funding is in fact the lowest funded category, surpassed by funding in the perinatal period, liver disease, congenital anomalies, and diabetes, all of which fall below the leading causes of death provisionally published by the CDC in 2023 [15]. This is also apparent in the leading causes of death published in 2019 by the CDC [14].

Transitions in CDC-funded categories from 2019 to 2022 somewhat parallel the leading causes of death, although direct comparison is difficult as the categories between the leading causes of death and CDC funding vary. Nevertheless, it can be argued that Injury Prevention and Control contribute more to the leading causes of death than HIV/AIDS, viral hepatitis, and STI and TB prevention since infectious disease is not a higher leading cause of death than trauma [14-16]. However, it is important to note that while CDC funding for Injury Prevention and Control has increased, this demarcation covers a wide range of topics. The “Urgent Threats” which constitute the primary focus of this budget category are Adverse Childhood Experiences (ACEs), Drug Overdose, and Suicide Prevention [17]. While these categories certainly impact the trauma field, they do not account for the magnitude and scope of traditional trauma research. Some effort and funding are allocated to drowning, transportation safety, older adult falls, and traumatic brain injuries. These are critical concerns for trauma research but are not prioritized by the Injury Prevention and Control budget.

NIH funding in relation to YPLL illustrates the extent to which trauma research is underfunded, especially when compared to the leading causes of death in the United States. The results of this study argue for the increased attention and funding of trauma research to help combat and reduce the morbidity and mortality associated with trauma. Research shows a mortality benefit when patients are treated by trauma system facilities rather than non-trauma facilities [18]. Moreover, level 1 trauma centers have better outcomes than level 2 trauma centers [19]. If improvement in trauma care is desired, then funding for trauma research should be a priority since research is a requirement by the American College of Surgeons to achieve a level 1 status [20].

The initiative to improve trauma research funding has not been stagnant. In 2014, The Coalition of National Trauma Research (CNTR), which has since created a National Trauma Research Action Plan (NTRAP) with the Department of Defense (DOD) in 2018 and a Scientific Advisory Council (SAC) in 2019, all with the goal of increasing funding in trauma research [5].

This study has limitations. Further analysis of every state would have provided more robust data to formulate conclusions. The difference in funding categories between the NIH and CDC as well as their differences in their “trauma” inclusion criteria also makes formulating comparisons challenging. Nevertheless, the ability to compare age-adjusted YPLL allows for accurate standardized comparisons between different states of different populations. 

## Conclusions

This study underscores a critical disparity in trauma research funding relative to its impact on public health and economic burden. Despite trauma being the leading cause of death and imposing a substantial economic strain annually in the US, trauma research remains significantly underfunded compared to other medical research fields. Our analysis reveals that the NIH allocates the smallest proportion of funding to trauma research per YPLL, with Oklahoma's funding levels being among the lowest when compared to other states.

Insufficient funding impedes advancements in trauma care and research, potentially compromising patient outcomes and straining healthcare resources. Enhanced research funding could facilitate improvements in trauma care, thereby reducing mortality rates and alleviating the associated healthcare burdens. Future research should focus on advocating for increased funding and exploring the potential impact of targeted investments in trauma research. This includes assessing the benefits of expanded trauma care systems and investigating the correlation between research funding and patient outcomes. Additionally, a broader, more detailed analysis across all states and funding agencies could provide a more comprehensive understanding of the funding landscape and its implications for trauma care. Addressing these gaps will be crucial for aligning research priorities with the pressing needs of trauma patients and improving overall healthcare delivery.
